# Coexpression Network Analysis of Macronutrient Deficiency Response Genes in Rice

**DOI:** 10.1186/s12284-015-0059-0

**Published:** 2015-07-24

**Authors:** Hinako Takehisa, Yutaka Sato, Baltazar Antonio, Yoshiaki Nagamura

**Affiliations:** Genome Resource Unit, National Institute of Agrobiological Sciences, 2-1-2 Kannondai, Tsukuba, Ibaraki 305-8602 Japan

**Keywords:** Coexpression analysis, Macronutrient deprivation, Rice, Transcriptome, Microarray

## Abstract

**Background:**

Macronutrients are pivotal elements for proper plant growth and development. Although extensive gene expression profiling revealed a large number of genes differentially expressed under various nutrient deprivation, characterization of these genes has never been fully explored especially in rice. Coexpression network analysis is a useful tool to elucidate the functional relationships of genes based on common expression. Therefore, we performed microarray analysis of rice shoot under nitrogen (N), phosphorus (P), and potassium (K) deficiency conditions. Moreover, we conducted a large scale coexpression analysis by integrating the data with previously generated gene expression profiles of organs and tissues at different developmental stages to obtain a global view of gene networks associated with plant response to nutrient deficiency.

**Results:**

We statistically identified 5400 differentially expressed genes under the nutrient deficiency treatments. Subsequent coexpression analysis resulted in the extraction of 6 modules (groups of highly interconnected genes) with distinct gene expression signatures. Three of these modules comprise mostly of downregulated genes under N deficiency associated with distinct functions such as development of immature organs, protein biosynthesis and photosynthesis in chloroplast of green tissues, and fundamental cellular processes in all organs and tissues. Furthermore, we identified one module containing upregulated genes under N and K deficiency conditions, and a number of genes encoding protein kinase, kinase-like domain containing protein and nutrient transporters. This module might be particularly involved in adaptation to nutrient deficiency via phosphorylation-mediated signal transduction and/or post-transcriptional regulation.

**Conclusions:**

Our study demonstrated that large scale coexpression analysis is an efficient approach in characterizing the nutrient response genes based on biological functions and could provide new insights in understanding plant response to nutrient deficiency.

**Electronic supplementary material:**

The online version of this article (doi:10.1186/s12284-015-0059-0) contains supplementary material, which is available to authorized users.

## Background

Nitrogen (N), phosphorus (P) and potassium (K) are essential macronutrients for plant growth and development playing important roles in various fundamental metabolic processes. Nutrient deficiencies associated with N, P, and K are abiotic stressors with major impact on plant growth that may eventually lead to serious agricultural yield losses. Genome-wide transcriptome profiling under nutrient deficiency conditions in model plants such as Arabidopsis (Hammond et al. 2003; Wu et al. 2003; Armengaud et al. 2004; Misson et al. 2005; Krapp et al. 2011) and rice (Lian et al. 2006; Wasaki et al. 2006; Ma et al. 2012; Takehisa et al. 2013) have identified a large number of genes specifically expressed in response to starvation to macronutrients. However, these nutrient deficiency response genes have never been fully characterized in terms of biological functions and molecular mechanisms particularly in rice. Coexpression network analysis is a powerful method to identify gene expression modules based on similarity of expression patterns and to predict the biological function of unknown genes. Indeed, this strategy has been demonstrated to uncover novel factors regulating specific metabolic pathways in Arabidopsis (Persson et al. 2005; Aoki et al. 2007; Hirai et al. 2007; Obayashi and Kinoshita, 2010). Therefore, we performed coexpression analysis using gene expression profiles derived not only under nutrient deficiency treatments but also expression data from various organs and tissues at different developmental stages in order to provide new insights into the biological function of responsive genes under nutrient deprivation in rice.

## Results and Discussion

### Identification of Nutrient Deficiency Response Genes

Seven-day old seedlings of rice (*Oryza sativa* L. ssp. *japonica* cultivar Nipponbare) were subjected to N, P and K deficiency treatments by hydroponic culture with each nutrient adjusted to 1/4, 1/16 and 1/64 of the normal concentration. After 5 days of nutrient deficiency treatments, shoot samples were collected and total RNA was extracted from each sample. Microarray analysis was performed using the Rice 4x44K Microarray RAP-DB (Agilent Technologies) platform which consists of 35,760 probes corresponding to 27,201 loci published in RAP-DB (Rice Annotation Project 2008; Sakai et al. 2013; Sato et al. 2011). We obtained a total of 36 microarray data corresponding to 12 samples with 3 replicates for each treatment and control conditions. All gene expression data are deposited in the NCBI Gene Expression Omnibus (Barrett et al. 2013, Accession no. GSE66935).

**Fig. 1:**
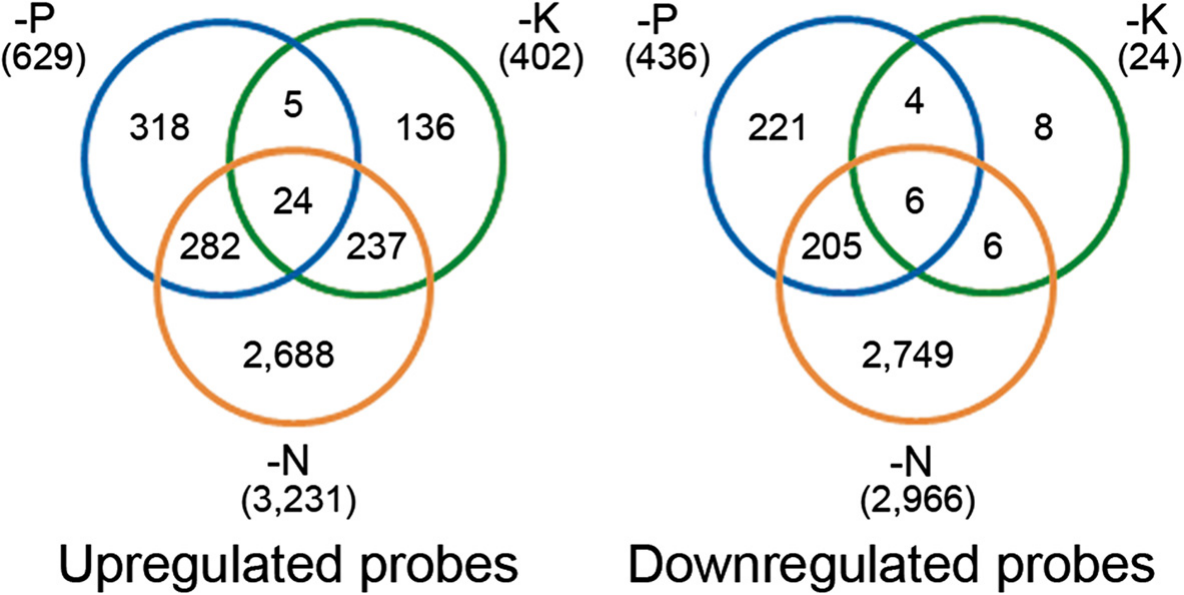
Venn diagram of upregulated and downregulated probes under N, P and K deficiency conditions. Differentially expressed probes were statistically extracted using *t*-test and fold change analysis (FDR < 0.05, FC > 2.0). The number in parenthesis indicates the number of all differentially expressed probes in each treatment

The raw signal intensity values of all probes were processed and subjected to 75 percentile normalization and log2 transformation. An additional normalization procedure was performed by subtracting the averaged signal intensity of the control (triplicate) from the value of each probe within each nutrient treatment condition. The normalized value was assigned as the relative expression value. A total of 28,402 probes corresponding to 21,282 loci with raw signal intensity above 100 in at least 3 of the 36 data were used in subsequent analysis. The differentially expressed probes were identified statistically using *t*-test (FDR < 0.05) and fold change analysis (FC > 2) in at least one of the 3 low nutrient condition (1/4, 1/16 and 1/64 of control concentration) against the control using the relative expression values. As a result, we obtained six probe sets corresponding to 3231 upregulated and 2966 downregulated probes under N deficiency (−N) condition, 629 upregulated and 436 downregulated probes under P deficiency (−P), and 402 upregulated and 24 downregulated probes under K deficiency (−K) condition. A large number of probes were differentially expressed under −N in shoot in comparison with −P and −K conditions as reported previously in root (Takehisa et al., 2013). Venn diagram analysis revealed 306 commonly upregulated probes under −N and −P, 261 commonly upregulated probes under −N and −K, and 211 commonly downregulated probes under −N and −P conditions (Fig. 1). A total of 6701 probes were differentially expressed in at least 1 of the 3 nutrient deficiency conditions (Additional file 1: Table S1). For genes with multiple probes, the average values of the probes were used. Overall, we obtained a total of 5400 genes which were defined as nutrient deficiency response genes (NRGs) in this paper.

### Coexpression Network Analysis of Nutrient Deficiency Response Genes

**Fig. 2:**
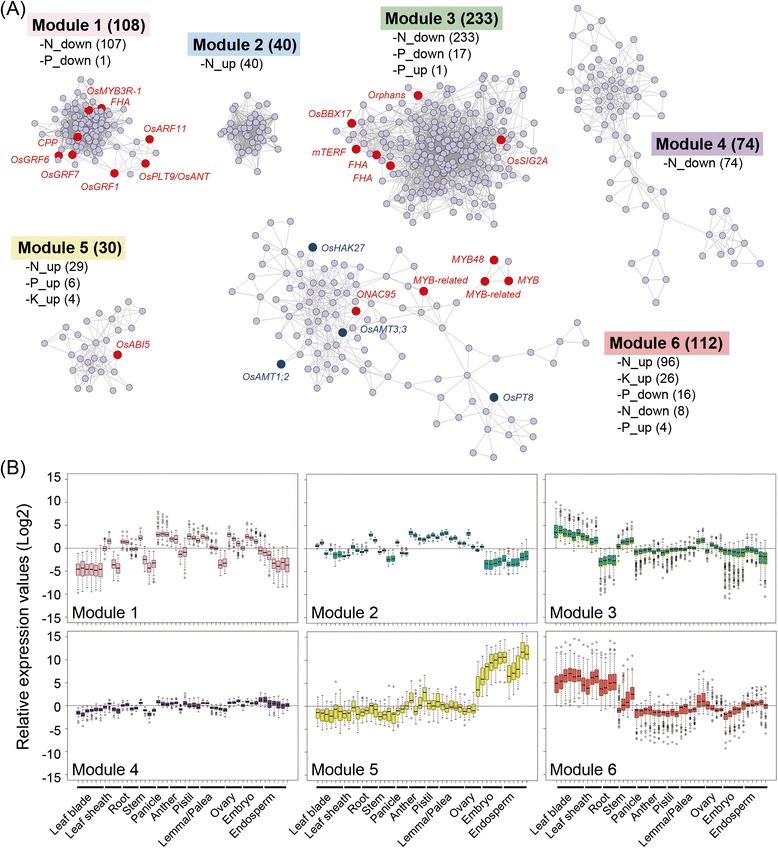
Coexpression network analysis of nutrient deficiency response genes (NRGs). **a** Six modules extracted from coexpression analysis using 179 microarray data. The number in parentheses indicates the number of NRGs in each module. Red and blue nodes indicate transcription factor and nutrient transporter genes, respectively. **b** Expression profile of NRGs in each module in various organs and tissues at different developmental stages based on 143 microarray data previously obtained by Sato et al. (2011) from leaf blade (6 samples), leaf sheath (4) root (4), stem (3), panicle (3), anther (3), pistil (3), lemma and palea (6), ovary (4), embryo (5), and endosperm (5). The vegetative organ samples were obtained mainly at the maturity stage of development, whereas almost all reproductive organ samples were obtained during the immaturity stage (Details in Additional file 2: Table S2). We performed 75 percentile normalization with log2 transformation and the relative expression value (log2) was obtained by subtracting the median expression value within the data set for each probe

Coexpression analysis was performed based on the expression profile of 5400 NRGs in 179 microarray data set. The data set consists of 36 data from N, P, and K deficiency treatments, and 143 data derived from previously generated gene expression profiles of various organs and tissues (GEO accession number: GSE21396; Sato et al. 2011; see Additional file 2: Table S2). We calculated the Pearson correlation coefficient (PCC) value for all pair-wise combinations of the 5400 NRGs and those PCC values above 0.9 were used for construction of the coexpression gene network (Additional file 3: Figure S1). Then using the MCODE program (degree cutoff: 2, node score cutoff: 0.9, K-core: 2, max depth: 100) in Cytoscape (Bader and Hogue 2003; Shannon et al. 2003), we were able to identify 17 coexpression modules in the gene network (Additional file 4: Table S3). Further cut-off with a cluster score > 3.0 resulted in 6 distinct modules with highly interconnected gene networks (Additional file 5: Table S4). Among them, module 2 consisted of 40 upregulated genes under -N condition (Fig. 2a). The probes designed for most of the module 2 genes seem to show unreliable expression because the probes of 33 out of the 40 genes were designed based on fragment sequences of full-length cDNA clones. In addition, 26 genes in module 2 encode unknown protein (Additional file 5; Table S4). Module 5 consisted of 30 genes upregulated under −N, −P and/or −K (Fig. 2a) including *ABA insensitive 5* (*OsABI5)* gene and *7* genes encoding late embryogenesis abundant protein (LEA) (Additional file 5; Table S4). It has been reported that the expression of *OsABI5* was induced by ABA and salt stress and that *OsABI5* was involved in the adaptive response to salt stress (Zou et al. 2008). The module 5 genes were expressed specifically in seed samples such as the embryo and endosperm, implying a probable role in seed development under normal conditions (Fig. 2b). Although the gene network is likely to be involved in response to nutrient deficiency, a more detailed analysis would be necessary to define the potential role of the genes identified in this module.

**Fig. 3:**
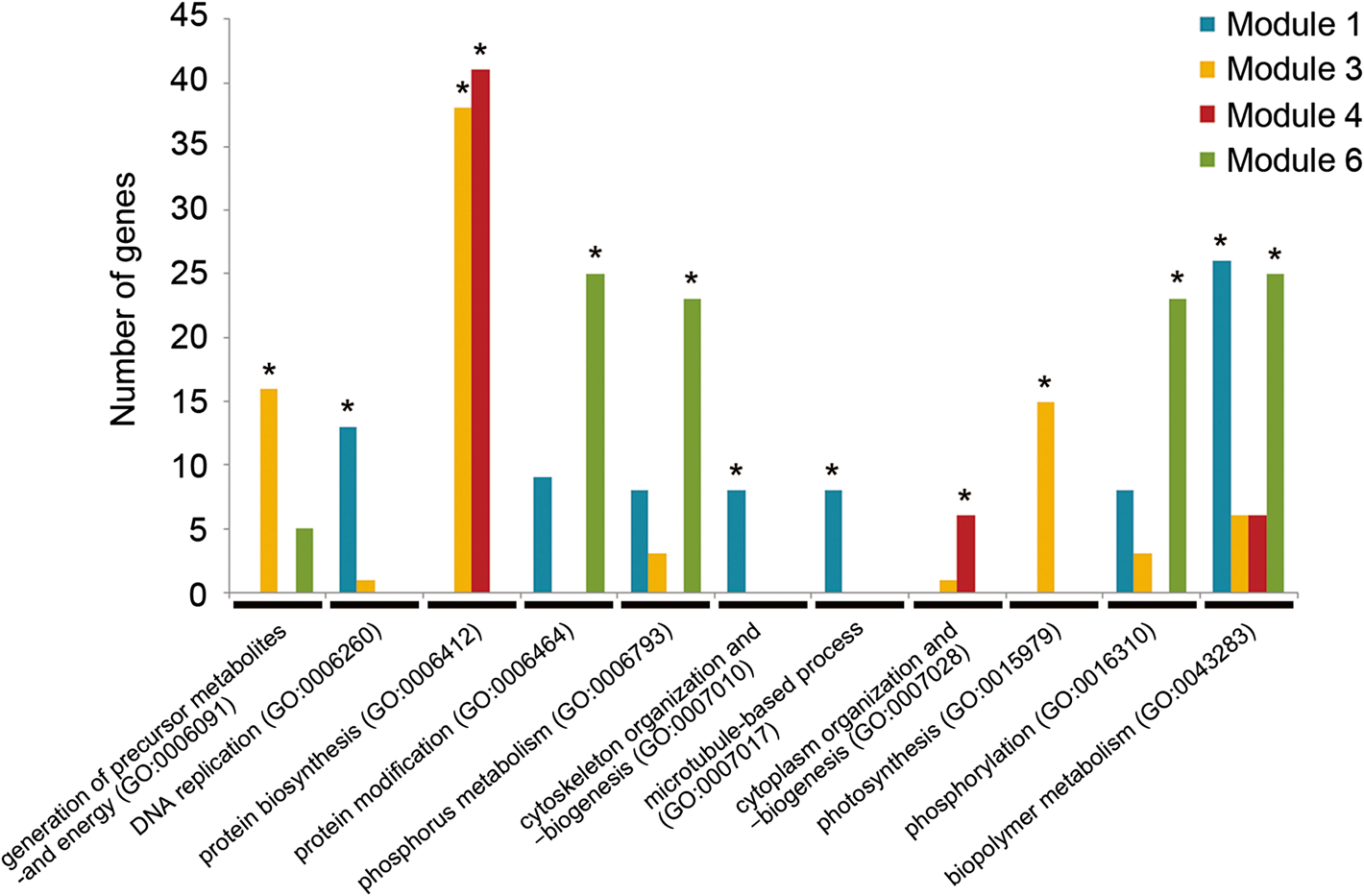
Gene ontology (GO) terms overrepresented in modules. Asterisks indicate significant overrepresented GO terms (FDR < 0.05, Number of genes > 5). The major enriched GO terms were indicated in the graph and all enriched terms were listed in Additional file 6: Table S5. There wear no enriched GO terms in module 2 and 5

Most of the genes in modules 1, 3 and 4 were downregulated under −N condition (Fig. 2a). However, GO enrichment analysis revealed distinct biological functions as follows: module 1 was significantly enriched with GO terms related to cell division and development such as ‘DNA replication (GO:0006260)’, ‘cytoskeleton organization and biosynthesis (GO:0007010)’, and ‘microtubule-based process (GO:0007017)’; module 3 was preferentially overrepresented with ‘protein biosynthesis (GO:0006412)’ and ‘photosynthesis (GO:0015979)’; and module 4 consisted mainly of ‘protein biosynthesis (GO:0006412)’ and ‘cytoplasm organization and biogenesis (GO:0007028)’ (Fig. 3, Additional file 6: Table S5). In module 1, we found 8 transcription factor genes, three of which encode growth-regulating factors (GRFs) (Fig. 2a). Some OsGRFs have known functions in plant growth and development, and strongly expressed in developing immature organs (Choi et al. 2004; Liu et al. 2014). Most genes included in module 3 encode chloroplast proteins such as 30S and 50S ribosomal proteins associated with protein biosynthesis in organelles, and chlorophyll biosynthesis related enzymes. In contrast, a number of genes encoding cytoplasmic 40S and 60S ibosomal proteins were grouped in module 4. Furthermore, the gene expression signatures in modules 1, 3, and 4 showed good coincidence with the predicted biological functions of each module (Fig. 2b). Module 1 genes were preferentially expressed in developing organs such as stem at early reproductive stage (before heading) and flower organs at various developmental stages. The genes of module 3 were highly expressed in vegetative organs such as leaf blade and leaf sheath. The module 4 genes were uniformly expressed in all organs and tissues. Taken together, the genes of modules 1, 3 and 4 are involved mainly in the development of immature organs, protein biosynthesis and photosynthesis in chloroplast of green tissues, and fundamental cellular process in all organs and tissues, respectively. It therefore appears that these functions are regulated directly and/or indirectly by the level of available nitrogen for proper plant growth.

In contrast, the majority of genes included in module 6 were upregulated under −N condition (Fig. 2a). This module also contains several genes differentially expressed under K and/or P deficiency. The GO terms corresponding to ‘protein modification (GO:0006464)’ and ‘phosphorylation (GO:0016310)’ were overrepresented with a large number of genes encoding protein kinase and kinase-like domain containing protein (Fig. 3, Additional file 5: Table S4). Previously, it has been reported that protein phosphorylation was significantly induced under the −N condition (Wang et al. 2012). More importantly, we found genes encoding various nutrient transporters such as ammonium (*OsAMT1;2* and *OsAMT3;3*), phosphate (*OsPT8*) and potassium (*OsHAK27*) transporters expressed preferentially in the leaf and root (Fig. 2). In Arabidopsis, a phosphorylation dependent regulation has been reported for transporters/channels related to transport of nitrate (NRT1;1/CHL1), potassium (AKT1) and ammonium (AMT1;1) under nutrient deficiency conditions (Liu and Tsay 2003; Lee et al. 2007; Loqué et al. 2007; Lanquar et al. 2009). Although it remains unclear if the protein kinase genes associated with module 6 directly and/or indirectly regulate the transporters via the phosphorylation machinery, the coexistence of many protein kinases and transporter genes in the same coexpression module provides new insights into the nutrient usage mechanism under nutrient deprivation conditions. We also noted that 4 out of 5 transcription factor genes included in module 6 were *MYB*-type. Several *MYB* genes such as *PHR* and *PAP* have been found to play important roles in the regulation of plant response and adaptation to nutrient deficiency (Rubio et al. 2001; Schachtman and Shin 2007; Feyissa et al. 2009; Tsay et al. 2011). Although these functional roles remain to be clarified, the *MYB* genes identified in this module may be involved in the adaptation to nutrient starvation.

Since module 6 contains genes expressed in response not only to −N but also to −P and −K (Fig. 2a), we further examined the relationships in gene expression signatures among the 3 nutrient deficiency conditions and found that the expression pattern between −N and −K showed positive correlation whereas the expression pattern between −K and −P showed negative correlation (Additional file 7: Figure S2). Previous studies suggest multiple relationships in response to N and K starvation, e.g., CIPK23 has been found to modulate the uptake activity of both the nitrate transporter, CHL1, and the potassium channel, AKT1, under nutrient deficiency conditions (Li et al. 2006; Xu et al. 2006; Ho et al. 2009). Therefore, the gene network identified in module 6 may be associated with crosstalk of N and K signaling as an adaptive response to nutrient starvation. On the other hand, we found very little evidence of interaction between P and K signaling, so further experiment-based analysis maybe be necessary to clarify the interaction between these macronutrients.

## Conclusions

Although we used whole shoot including mature and immature leaves for gene expression profiling under nutrient deficiency conditions, a large scale coexpression analysis enabled us to define distinct modules with specific biological functions. Recently, gene expression data covering various experimental conditions have increased significantly and could be easily accessed in the public domain such as NCBI-GEO (Barrett et al. 2013) and ArrayExpress (Kolesnikov et al. 2015). Therefore, transcriptome analysis aimed at elucidating the complex gene networks involved in response to nutrient starvation could be further enhanced by large-scale coexpression analysis using specific expression data, thereby providing a network-level understanding of adaptive mechanisms to nutrient deficiency. We identified a module which may be associated with direct nutrient transport, signal transduction and regulation in nutrient deficiency conditions in rice. Further detailed analysis of expressed genes and associated gene networks may provide new insights into the molecular mechanism of nutrient usage with direct ramifications in the growth and development of the rice plant.

## Methods

### Plant Material and Nutrient Deficiency Treatment Conditions

Rice (*Oryza sativa* L. ssp. *japonica* cultivar Nipponbare) seeds were sterilized with 70 % ethanol solution and 1 % sodium hypochlorite solution, imbibed in distilled water in the dark at 28 °C for 2 days. The germinated seeds were transferred onto a nylon net floated in distilled water in a growth chamber (60 % humidity; 14-h light at 28 °C and 10-h dark at 25 °C). After 3 days, the seedlings were transferred to a normal nutrient solution (5-fold dilution of Yoshida’s nutrient solution; pH 5.5; Yoshida et al. 1976). Seven-day old seedlings were subjected to N, P, and K deficiency treatments, respectively. The amounts of NH_4_NO_3_ for N deficiency treatment, NaH_2_PO_4_・2H_2_O for P deficiency treatment, and K_2_SO_4_ for K deficiency treatment were changed to adjust deficiency levels (1/64, 1/16, 1/4) of each nutrient, respectively. The concentrations of NH_4_NO_3_, NaH_2_PO_4_・2H_2_O, and K_2_SO_4_ in control conditions were 0.285, 0.065 and 0.102 mM, respectively. Shoot samples (PO:0009006; shoot system, PO:0007112; 1 main shoot growth stage, Cooper et al. 2013) were collected at 5 days after nutrient starvation. The pH of the nutrient solution was adjusted using 1 N NaOH and maintained with 2-(N-morpholine)-ethanesulphonic acid MES buffer. The solution was renewed every 2 days.

### RNA Extractions and Microarray Analysis

Total RNA was extracted from each sample with RNeasy Plant Mini kit (QIAGEN) according to the manufacturer’s protocol. The quantity and quality of the obtained RNA was checked with Agilent 2100 Bioanalyzer (Agilent Technologies, Palo Alto, CA, USA). Labeling and hybridization on a slide of rice 4x44K microarray RAP-DB (Agilent; G2519F#15241) was performed with one-color method according to Sato et al. 2011. Slides were scanned on an Agilent G2505C DNA microarray scanner. The scanned images were analyzed with Feature Extraction Software 10.5.1.1 (Agilent) using default parameters to obtain background subtracted and spatially detrended processed signal intensities.

### Statistical Analysis

The processed raw signal intensity of all probes (45,151) was applied to 75 percentile normalization and log2 transformation. The averaged values of the 3-replicate control samples were subtracted for each probe within each nutrient treatment condition. The normalization procedure was performed using GeneSpring GX12 software (Agilent Technologies) and the normalized signal intensity was designated as relative expression value. A total of 35,760 probes were extracted and used in statistical test. t-test, fold change analysis and Venn diagram analysis were also performed using GeneSpring GX12.

### Coexpression Analysis

Coexpression analysis was performed based on the expression profile of 5400 NRGs in 179 microarray data corresponding to 36 data from N, P, and K deficiency treatments, and 143 data derived from previous transcriptome analysis of various organs and tissues. We performed 75 percentile normalization with log2 transformation. For the nutrient deficiency treatment data, the average value of the 3 control samples was subtracted for each probe within each nutrient. For the organ and tissue transcriptome data, the median expression value within each data set was subtracted for each probe. The expression values of 5400 genes were extracted after combining the two expression data sets and getting the average value for loci with multiple probes. The calculation of Pearson correlation coefficient was performed with the R program (R Development Core Team, 2011).

### GO Enrichment Analysis

GO enrichment test was performed against terms of biological process using the option available in the gene coexpression database RiceFREND (Sato et al. 2013). We extracted significant overrepresented GO terms with FDR < 0.05 and number of genes > 5.

### Resources for Gene Annotation

The gene annotation was obtained from the RAP-DB (Rice Annotation Project, 2008), PLANT TRANSCRIPTION FACTOR DATABASE (Pérez-Rodríguez et al. 2010), and Oryzabase (Kurata and Yamazaki 2006).

### Data Access

The data used in this study was deposited in NCBI-GEO (Barrett et al. 2013) and is accessible through GSE66935 for the gene expression profiles under nutrient deficiency and GSE21396 for the profiles of various organs and tissues (Sato et al. 2011).

## Additional files

Additional file 1: Table S1. List of differentially expressed probes in rice shoot under nitrogen (N), phosphorus (P) and potassium (K) deficiency conditions. Additional file 2: Table S2. List of microarray data of various organs and tissues. Additional file 3: Figure S1. Pearson correlation coefficient cutoff determination. Additional file 4: Table S3. List of coexpression modules identified by MCODE. Additional file 5: Table S4. List of nutrient deficiency response genes (NRGs) identified in the six modules. Additional file 6: Table S5. List of enriched GO terms in the coexpression modules. Additional file 7: Figure S2. Relationships of the expression signatures of genes in module 6 under nutrient deficiency conditions.

## Abbreviations

FDR: False discovery rate
FC: Fold change
GEO: Gene expression omnibus
GO: Gene ontology
NRGs: Nutrient deficiency response genes
PCC: Pearson correlation coefficient

## References

[CR1] Aoki K, Ogata Y, Shibata D (2007). Approaches for extracting practical information from gene co-expression networks in plant biology. Plant Cell Physiol.

[CR2] Armengaud P, Breitling R, Amtmann A (2004). The potassium-dependent transcriptome of Arabidopsis reveals a prominent role of jasmonic acid in nutrient signaling. Plant Physiol.

[CR3] Bader GD, Hogue CW (2003). An automated method for finding molecular complexes in large protein interaction networks. BMC Bioinf.

[CR4] Barrett T, Wilhite SE, Ledoux P, Evangelista C, Kim IF, Tomashevsky M, Marshall KA, Phillippy KH, Sherman PM, Holko M, Yefanov A, Lee H, Zhang N, Robertson CL, Serova N, Davis S, Soboleva A (2013). NCBI GEO: archive for functional genomics data sets-update. Nucleic Acids Res.

[CR5] Choi D, Kim JH, Kende H (2004). Whole genome analysis of the *OsGRF* gene family encoding plant-specific putative transcription activators in rice (*Oryza sativa* L.). Plant Cell Physiol.

[CR6] Cooper L, Walls RL, Elser J, Gandolfo MA, Stevenson DW, Smith B, Preece J, Athreya B, Mungall CJ, Rensing S, Hiss M, Lang D, Reski R, Berardini TZ, Li D, Huala E, Schaeffer M, Menda N, Arnaud E, Shrestha R, Yamazaki Y, Jaiswal P (2013). The Plant Ontology as a tool for comparative plant anatomy and genomic analyses. Plant Cell Physiol.

[CR7] Feyissa DN, Løvdal T, Olsen KM, Slimestad R, Lillo C (2009). The endogenous *GL3*, but not *EGL3*, gene is necessary for anthocyanin accumulation as induced by nitrogen depletion in *Arabidopsis* rosette stage leaves. Planta.

[CR8] Hammond JP, Bennett MJ, Bowen HC, Broadley MR, Eastwood DC, May ST, Rahn C, Swarup R, Woolaway KE, White PJ (2003). Changes in gene expression in Arabidopsis shoots during phosphate starvation and the potential for developing smart plants. Plant Physiol.

[CR9] Hirai MY, Sugiyama K, Sawada Y, Tohge T, Obayashi T, Suzuki A, Araki R, Sakurai N, Suzuki H, Aoki K, Goda H, Nishizawa OI, Shibata D, Saito K (2007). Omics-based identification of *Arabidopsis* Myb transcription factors regulating aliphatic glucosinolate biosynthesis. Proc Natl Acad Sci U S A.

[CR10] Ho CH, Lin SH, Hu HC, Tsay YF (2009). CHL1 functions as a nitrate sensor in plants. Cell.

[CR11] Kolesnikov N, Hastings E, Keays M, Melnichuk O, Tang YA, Williams E, Dylag M, Kurbatova N, Brandizi M, Burdett T, Megy K, Pilicheva E, Rustici G, Tikhonov A, Parkinson H, Petryszak R, Sarkans U, Brazma A (2015). ArrayExpress update-simplifying data submissions. Nucleic Acids Res.

[CR12] Krapp A, Berthomé R, Orsel M, Mercey-Boutet S, Yu A, Castaings L, Elftieh S, Major H, Renou JP, Daniel-Vedele F (2011). Arabidopsis roots and shoots show distinct temporal adaptation patterns toward nitrogen starvation. Plant Physiol.

[CR13] Kurata N, Yamazaki Y (2006). Oryzabase. An integrated biological and genome information database for rice. Plant Phsiol.

[CR14] Lanquar V, Loqué D, Hörmann F, Yuan L, Bohner A, Engelsberger WR, Lalonde S, Schulze WX, von Wirén N, Frommer WB (2009). Feedback inhibition of ammonium uptake by a phospho-dependent allosteric mechanism in Arabidopsis. Plant Cell.

[CR15] Lee SC, Lan WZ, Kim BG, Li L, Cheong YH, Pandey GK, Lu G, Buchanan BB, Luan S (2007). A protein phosphorylation/dephosphorylation network regulates a plant potassium channel. Proc Natl Acad Sci U S A.

[CR16] Li L, Kim BG, Cheong YH, Pandey GK, Luan S (2006). A Ca^2+^ signaling pathway regulates a K^+^ channel for low-K response in *Arabidopsis*. Proc Natl Acad Sci U S A.

[CR17] Lian X, Wang S, Zhang J, Feng Q, Zhang L, Fan D, Li X, Yuan D, Han B, Zhang Q (2006). Expression profiles of 10,422 genes at early stage of low nitrogen stress in rice assayed using a cDNA microarray. Plant Mol Biol.

[CR18] Liu KH, Tsay YF (2003). Switching between the two action modes of the dual-affinity nitrate transporter CHL1 by phosphorylation. EMBO J.

[CR19] Liu H, Guo S, Xu Y, Li C, Zhang Z, Zhang D, Xu S, Zhang C, Chong K (2014). OsmiR396d-regulated OsGRFs function in floral organogenesis in rice through binding to their targets *OsJMJ706* and *OsCR4*. Plant Physiol.

[CR20] Loqué D, Lalonde S, Looger LL, von Wirén N, Frommer WB (2007). A cytosolic trans-activation domain essential for ammonium uptake. Nature.

[CR21] Ma TL, Wu WH, Wang Y (2012). Transcriptome analysis of rice root responses to potassium deficiency. BMC Plant Biol.

[CR22] Misson J, Raghothama KG, Jain A, Jouhet J, Block MA, Bligny R, Ortet P, Creff A, Somerville S, Rolland N, Doumas P, Nacry P, Herrerra-Estrella L, Nussaume L, Thibaud MC (2005). A genome-wide transcriptional analysis using *Arabidopsis thaliana* Affymetrix gene chips determined plant responses to phosphate deprivation. Proc Natl Acad Sci U S A.

[CR23] Obayashi T, Kinoshita K (2010). Coexpression landscape in ATTED-II: usage of gene list and gene network for various types of pathways. J Plant Res.

[CR24] Pérez-Rodríguez P, Riaño-Pachón DM, Corrêa LG, Rensing SA, Kersten B, Mueller-Roeber B (2010). PlnTFDB: updated content and new features of the plant transcription factor database. Nucleic Acids Res.

[CR25] Persson S, Wei H, Milne J, Page GP, Somerville CR (2005). Identification of genes required for cellulose synthesis by regression analysis of public microarray data sets. Proc Natl Acad Sci U S A.

[CR26] Development Core Team R (2011). R: A language and environment for statistical computing R Foundation for Statistical Computing Vienna R Development Core Team (2011) R: A language and environment for statistical computing.

[CR27] Rice Annotation Project (2008). The Rice Annotation Project Database (RAP-DB): 2008 update. Nucleic Acids Res.

[CR28] Rubio V, Linhares F, Solano R, Martin AC, Iglesias J, Leyva A, Paz-Ares J (2001). A conserved MYB transcription factor involved in phosphate starvation signaling both in vascular plants and in unicellular algae. Genes Dev.

[CR29] Sakai H, Lee SS, Tanaka T, Numa H, Kim J, Kawahara Y, Wakimoto H, Yang CC, Iwamoto M, Abe T, Yamada Y, Muto A, Inokuchi H, Ikemura T, Matsumoto T, Sasaki T, Itoh T (2013). Rice Annotation Project Database (RAP-DB): an integrative and interactive database for rice genomics. Plant Cell Physiol.

[CR30] Sato Y, Antonio BA, Namiki N, Motoyama R, Sugimoto K, Takehisa H, Minami H, Kamatsuki K, Kusaba M, Hirochika H, Nagamura Y (2011). Field transcriptome revealed critical developmental and physiological transitions involved in the expression of growth potential in *japonica* rice. BMC Plant Biol.

[CR31] Sato Y, Namiki N, Takehisa H, Kamatsuki K, Minami H, Ikawa H, Ohyanagi H, Sugimoto K, Itoh J, Antonio B, Nagamura Y (2013). RiceFREND: a platform for retrieving coexpressed gene networks in rice. Nucleic Acids Res.

[CR32] Schachtman DP, Shin R (2007). Nutrient sensing and signaling: NPKS. Annu Rev Plant Biol.

[CR33] Shannon P, Markiel A, Ozier O, Baliga NS, Wang JT, Ramage D, Amin N, Schwikowski B, Ideker T (2003). Cytoscape: a software environment for integrated models of biomolecular interaction networks. Genome Res.

[CR34] Takehisa H, Sato Y, Antonio BA, Nagamura Y (2013). Global transcriptome profile of rice root in response to essential macronutrient deficiency. Plant Signal Behav.

[CR35] Tsay YF, Ho CH, Chen HY, Lin SH (2011). Integration of nitrogen and potassium signaling. Annu Rev Plant Biol.

[CR36] Wang X, Bian Y, Cheng K, Zou H, Sun SSM, He JX (2012). A comprehensive differential proteomic study of nitrate deprivation in *Arabidopsis* reveals complex regulatory networks of plant nitrogen responses. J Proteome Res.

[CR37] Wasaki J, Shinano T, Onishi K, Yonetani R, Yazaki J, Fujii F, Shimbo K, Ishikawa M, Shimatani Z, Nagata Y, Hashimoto A, Ohta T, Sato Y, Miyamoto C, Honda S, Kojima K, Sasaki T, Kishimoto N, Kikuchi S, Osaki M (2006). Transcriptomic analysis indicates putative metabolic changes caused by manipulation of phosphorus availability in rice leaves. J Exp Bot.

[CR38] Wu P, Ma L, Hou X, Wang M, Wu Y, Liu F, Deng XW (2003). Phosphate starvation triggers distinct alterations of genome expression in Arabidopsis roots and leaves. Plant Physiol.

[CR39] Xu J, Li HD, Chen LQ, Wang Y, Liu LL, He L, Wu WH (2006). A protein kinase, interacting with two calcineurin B-like proteins, regulates K^+^ transporter AKT1 in *Arabidopsis*. Cell.

[CR40] Yoshida S, Forno DA, Cook JH, Gomez KA, Yoshida S, Forno DA, Cook JH, Gomez KA (1976). Routine procedures for growing rice plants in culture solution. Laboratory Manual for Physiological Studies of Rice.

[CR41] Zou M, Guan Y, Ren H, Zhang F, Chen F (2008). A bZIP transcription factor, OsABI5, is involved in rice fertility and stress tolerance. Plant Mol Biol.

