# Supplementary material for: Coexpression Network Analysis of Macronutrient Deficiency Response Genes in Rice
Source: Rice (N Y). 2015 Jul 24;8:24. doi: 10.1186/s12284-015-0059-0 (PMC4513034; doi:10.1186/s12284-015-0059-0)
Supplement: Additional file 3: Figure S1. — Pearson correlation coefficient cutoff determination. [file 12284_2015_59_MOESM3_ESM.pdf]

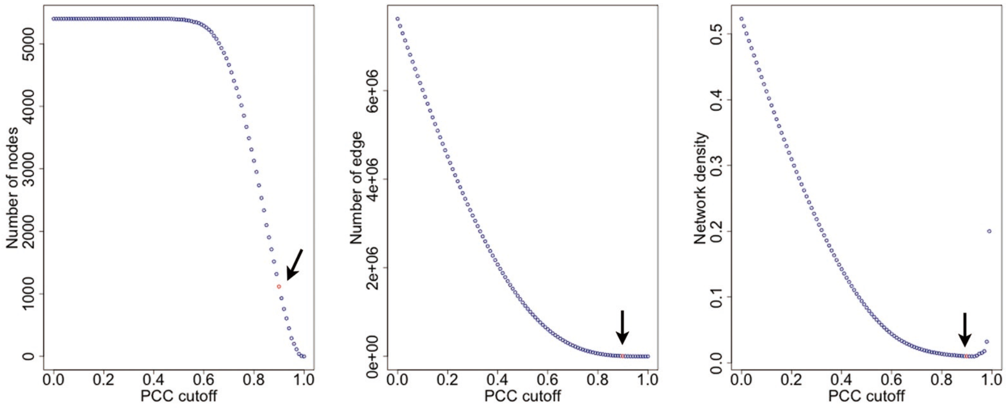

Additional file 3: Figure S1. Pearson correlation coefficient cutoff determination. The number of nodes (left), number of edges (center) and network density (right) at different PCC cutoff values are shown with the accepted cutoff value (PCC>0.9) indicated by arrows. The calculation was performed based on the expression profile of 5,400 NRGs in 179 microarray data corresponding to the 2 data sets, i.e., 36 data from N, P, and K deficiency treatments and 143 data derived from previous transcriptome analysis of various organs and tissues. We performed 75 percentile normalization with log2 transformation. For the nutrient deficiency treatment data, the averaged value of the 3 control samples were subtracted for each probe within each nutrient. For the organ and tissue trasnscriptome data, the median expression value within the data set was subtracted for each probe. The expression values of 5,400 genes were extracted after combining the two expression data and getting the average value for loci with multiple probes.
