# Supplementary material for: Coexpression Network Analysis of Macronutrient Deficiency Response Genes in Rice
Source: Rice (N Y). 2015 Jul 24;8:24. doi: 10.1186/s12284-015-0059-0 (PMC4513034; doi:10.1186/s12284-015-0059-0)
Supplement: Additional file 7: Figure S2. — Relationships of the expression signatures of genes in module 6 under nutrient deficiency conditions. [file 12284_2015_59_MOESM7_ESM.pdf]

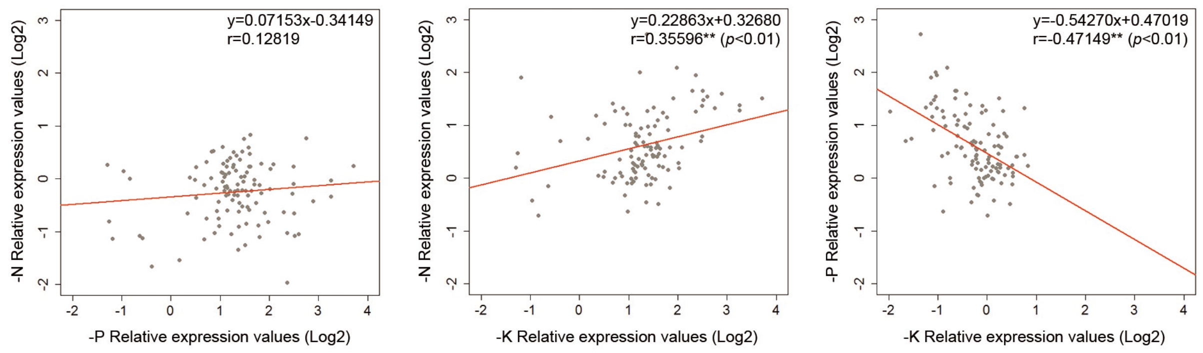

Additional file 7: Figure S2. Relationships of the expression signatures of genes in module 6 under nutrient deficiency conditions. The scatter plot was constructed using the values of log2 treatment/control in the 1/16 nitrogen, phosphorus and potassium treatment conditions. Red lines indicate regression line.
